# Mapping the Minnesota Living with Heart Failure Questionnaire (MLHFQ) to SF-6Dv2 in Chinese patients with heart failure

**DOI:** 10.1186/s12955-022-02004-x

**Published:** 2022-06-20

**Authors:** Jianni Cong, Yanbo Zhu, Jinhang Du, Lin Lin, Yuan He, Qian Zhang, Tan Ooh Chye, Xiaoying Lv, Wenqiong Liu, Xinrui Wu, Fanghui Ma, Xinyuan Zhao, Yuqiong Li, Liqun Long

**Affiliations:** 1 School of Chinese Medicine, Beijing University of Chinese Medicine, Beijing, 102488 China; 2 School of Management, Beijing University of Chinese Medicine, Beijing, 102488 China; 3 Cardiology Department of Integrated Traditional Chinese and Western Medicine, China-Japan Friendship Hospital, Beijing, 100029 China; 4 Department of Personnel, China-Japan Friendship Hospital, Beijing, 100029 China; 5 University of Paris Saclay, 91190 Saint-Aubin, France

**Keywords:** MLHFQ, SF-6Dv2, Heart failure, Utility, Mapping

## Abstract

**Purpose:**

Mapping the Minnesota Living with Heart Failure Questionnaire (MLHFQ) to SF-6Dv2 in Chinese patients with chronic heart failure, and to obtain the health utility value for health economic assessment.

**Methods:**

Four statistical algorithms, including ordinary least square method (OLS), Tobit model, robust MM estimator (MM) and censored least absolute deviations (CLAD), were used to establish the alternative model. Models were validated by using a tenfold cross-validation technique. The mean absolute error (MAE) and root mean square error (RMSE) were used to evaluate the prediction performance of the model. The Spearman correlation coefficient and Intraclass Correlation Coefficients (ICC) were used to examine the relationship between the predicted and observed SF-6Dv2 values.

**Results:**

A total of 195 patients with chronic heart failure were recruited from 3 general hospitals in Beijing. The MLHFQ summary score and domain scores of the study sample were negatively correlated with SF-6Dv2 health utility value. The OLS regression model established based on the MLHFQ domain scores was the optimal fitting model and the predicted value was highly positively correlated with the observed value.

**Conclusion:**

The MLHFQ can be mapped to SF-6Dv2 by OLS, which can be used for health economic assessment of cardiovascular diseases such as chronic heart failure.

**Supplementary Information:**

The online version contains supplementary material available at 10.1186/s12955-022-02004-x.

## Background

Heart failure (HF) is a major public health problem affecting at least 26 million patients worldwide [1]. The prevalence of HF in the United States alone is about 5.7 million, and there are about 670,000 new cases annually. In Europe, HF resulted in more than 1 million hospitalizations annually [2]. In China, the morbidity of HF in adults over 35 years old is about 1.3%, which affects more than 13 million patients [3]. The incidence and prevalence of heart failure are age dependent [4], as the population ages, the number of patients affected by HF will continue to increase. HF is associated with enormous health care expenditures and poses a huge economic burden to the health budgets globally. The annual cost of heart failure ranges from $ 908 to $ 40,971 per patient [4]. The overall economic cost of HF was estimated to be$108 billion per annum globally and will continue to rise [5]. Due to the considerable cost impact of HF on the healthcare system, it is necessary to optimize the disease management plan and carry out health economic assessment [6].

Health economic assessment plays an important role for making health decisions and optimizing health care resource allocation. It can help healthcare professionals and policy makers to select appropriate interventions for different patients and different health care setting. Cost utility analysis is one method of health economic assessment [7]. Quality adjusted life years (QALYs) which combine the quantity of life and quality of life is an important indicator needed to carry out cost utility analysis. A measurement instrument based on health preferences can obtain the health utility value needed to calculate QALYs [8].

Minnesota Living with Heart Failure Questionnaire (MLHFQ) is the most used instrument to evaluate the quality of life in patients with heart failure [9–11]. MLHFQ captures the symptom of HF and patients’ concerns, such as swelling, hospitalization, breathing, and so on. However, MLHFQ is not an instrument based on the measurement of health preferences, and the health utility value in patients with HF cannot be obtained. When we cannot obtain appropriate health utility data, mapping is regarded as a "second best" solution [12]. Mapping can estimate the relationship between the preference-based instrument and non-preference-based instrument by establishing statistical association. So mapping can transform the scale information of non-preference-based instrument into the health utility value based on preference-based instrument [13]. Britain’s National Institute for Health and Clinical Excellence (NICE) pointed out: "when there is no health utility value data or data is improper, mapping can be considered from other health related quality of life data, thereby gaining the right health utility value" in its guide of health economics evaluation methods [14].

SF-6D, including SF-6Dv1 and SF-6Dv2, is a globally used instrument based on health preferences [15, 16]. It can help us obtain health utility value for cost utility analysis. To the author’s knowledge, there is no study about mapping algorithm from MLHFQ to SF-6Dv1or SF-6Dv2 currently. The aim of this study was to try and develop the mapping algorithm which can construct a transformation model from MLHFQ to SF-6Dv2 and generate the health utility value for cost utility analysis in cardiovascular diseases such as CHF. Besides saving time and cost, it also provides health utility value which strongly correlates with the disease.

## Methods

### Study design

The study was based on the previous prospective study (the cost-effectiveness analysis methodology study in the treatment of CHF with traditional Chinese medicine), and the details of clinical study design which were reported in other literatures [17]. 199 patients were enrolled in the study who were diagnosed with CHF. They were recruited from inpatient departments of three general hospitals in Beijing from September 2009 to December 2011. Four cases of them were not included in the analysis due to incomplete information. All eligible patients signed the written informed consent and satisfied with the New York Heart Association (NYHA) I to IV. Patients with mental illness, congenital heart disease or severe impairment of liver and kidney function were excluded. Pregnant patients, patients with allergies and patients with acute myocardial infarction or unstable angina pectoris within the last month were also excluded. All eligible patients were surveyed twice before and 2 weeks after the survey, and one case was lost due to failure to follow-up.

### Instruments

#### Minnesota Living with Heart Failure Questionnaire

MLHFQ is a specific instrument for self-assessment quality of life on patients with heart failure, with good reliability and validity [18]. MLHFQ contains 21 items with 0–5 Likert instrument. Scores range from 0 to 105, representing the best to worst quality of life. MLHFQ has two domains, the physical domain (8 items, score range 0–40) and the emotional domain (5 items, score range 0–25), with the remaining 8 items only been used to calculate the total score [19]. MLHFQ is widely used in various countries [20–22] and has been translated into Chinese language and verified [23].

### SF-6Dv2

SF-6D is a health utility measurement instrument developed by Brazier [24] based on the Short Form-36 (SF-36). SF-6D instrument has two version, SF-6Dv1 and SF-6Dv2. SF-6Dv2 is an improved version of SF-6Dv1 with more distinct levels of health and clearer descriptions. SF-6Dv2 is derived from 10 items of SF-36v2, with a total of 6 dimensions, namely physical function (5 levels), role limitation (5 levels), social function (5 levels), pain (6 levels), mental health (5 levels) and vitality (5 levels), which can be used to describe 18,750 health states [25].

SF-6Dv2 can be used as a dependent or an independent instrument in three different ways. As dependent instrument, SF-6Dv2 can be combined with the full SF-36v2 (SF-6Dv2_SF-36_). As independent instrument, SF-6Dv2 can be used with only 10 items from the SF-36v2 (SF-6Dv2_ind-10_) or with 6 questions rephrased from 10 items of SF-36v2 (SF-6Dv2_ind-6)_. The three ways have similar results, with a high degree of consistency [26].

In this study, SF-6Dv2 combined with the full SF-36v2 (SF-6Dv2_SF-36_) was used to obtain the health state of the CHF patients. The Chinese-specific value set for the SF-6Dv2 range from − 0.277 to 1 [27].

### Training and testing sets

Using computer generated random numbers, the full sample (baseline and follow-up) were divided into two groups. 80% of the data (311 cases) were assigned to the training set with 20% (78 cases) of testing set. A tenfold cross-validation technique was employed to validate our models in training set. Goodness-of-fit of the models were evaluated by the average mean absolute error (MAE) and root mean square error (RMSE) from tenfold cross-validation. The smaller MAE and RMSE are, the better goodness-of-fit will be. Then all the data of training set were used to build the best model possible. Finally, the model was tested with the testing set.

### Statistical analysis

In the study, Shapiro–Wilk test and scatter chart were used to evaluate the normality of continuous variables, and spearman correlation and scatter plots were used to test the correlation between the MLHFQ score and the health utility value of SF-6Dv2. The SF-6Dv2 health utility value was taken as the dependent variable and the MLHFQ instrument information as the independent variable in the regression analysis. To detect the nonlinear relationship between the health utility value of SF-6Dv2 and the information of MLHFQ instrument, and to improve the prediction accuracy of the model, square terms and interaction terms were included in the models. Age and gender were included into the models to evaluate whether general demographic characteristics affected the relationship between SF-6Dv2 health utility value and MLHFQ instrument information. The specific models contained two aspects:

- Model A Main effect (total MLHFQ score + total MLHFQ score^2^) + covariate (sex, age)
- Model B Main effect (physical domain score + emotional domain score + remainder items score + physical domain score^2^ + emotional domain score^2^ + physical domain score * emotional domain score) + covariate (sex and age)

The models were estimated by ordinary least squares (OLS), Tobit model, robust MM estimator (MM) and censored least absolute deviations (CLAD). The relationship between the predicted and observed SF-6Dv2 values was examined by the Intraclass Correlation Coefficients (ICC). The consistency of health state was assessed by Bland–Altman plot. Scatter plots and spearman correlation were also presented.

All statistical analyses were performed using STATA 15.1 software.

## Results

### Sample characteristics

A total of 195 patients with CHF participated in the study, one of them was lost from follow-up and 194 patients were followed up. The characteristics of the sample is shown in Table 1. The mean age of the study participants was 69 years, ranged from 27 to 88. The sex ratio was about the same. The cardiac function classification of NYHA was mainly grade II and III in the baseline sample, and mainly I and II in the follow-up sample. The mean values of SF-6Dv2 in baseline and follow-up samples were 0.449 (SD = 0.324) and 0.649 (SD = 0.172), respectively. The mean of MLHFQ in baseline and follow-up samples were 49.969 (SD = 26.497) and 33.227 (SD = 21.511), respectively. The health status of the patients improved after intervention. The differences in MLHFQ total score, physical and emotional scores and SF-6Dv2 are reported in Additional file 1: Table S1.

**Table 1:** Patient characteristics

	Baseline sample		Follow-up sample	
	N = 195	Range	N = 194	Range
Male, n (%)	98 (50.26)		97 (50.00)	
Age, mean (SD)	69.51 ± 11.67	27–88	69.48 ± 11.69	27–88
NYHA, n (%)				
I	2 (1.03)		39 (20.10)	
II	52 (26.67)		122 (62.89)	
III	105 (53.85)		28 (14.43)	
IV	36 (18.46)		5 (2.58)	
MLHFQ, mean (SD)	49.969 ± 26.497	0–103	33.227 ± 21.511	0–102
Physical, mean (SD)	23.882 ± 12.191	0–40	15.103 ± 10.031	0–40
Emotion, mean (SD)	9.851 ± 6.96	0–25	6.428 ± 5.300	0–25
SF-6Dv2, mean (SD)	0.449 ± 0.324	− 0.269 to 0.962	0.649 ± 0.172	0.124–0.962

### Distribution of MLHFQ and SF-6Dv2

**Fig. 1:**
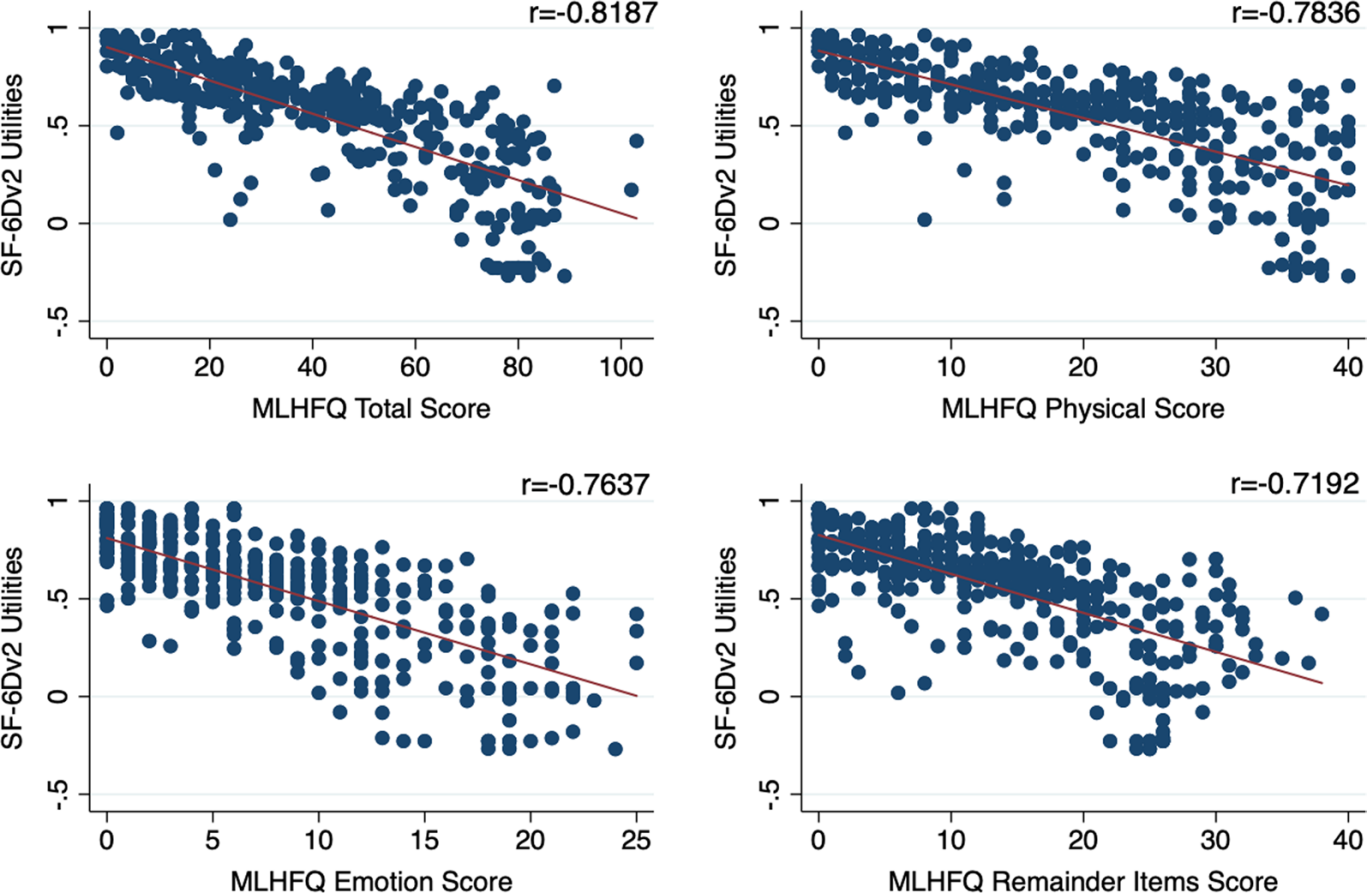
The correlation of MLHFQ scores with SF-6Dv2 utilities in full sample

By Shapiro–Wilk test, the total MLHFQ score and the SF-6Dv2 health utility values in the full samples were not normally distributed (*P* < 0.001). Figure 1 presents that the MLHFQ scores in different domains are negatively correlated with the health utility value of SF-6Dv2 in full sample. Spearman correlation results further verified the correlation. Correlation coefficients of total MLHFQ scores, physical domain scores, emotion domain scores and remainder items scores with health utility value of SF-6Dv2 were − 0.8187, − 0.7836, − 0.7637 and − 0.7192, respectively, all showing high negative correlation.

The correlation between dimensions of SF-6Dv2 and the MLHFQ are reported in Additional file 1: Table S2.

### Goodness-of-fit results of training sets

Table 2 presents the goodness-of-fit results of different models with four statistical algorithms from tenfold cross-validation. The details can be seen in Additional file 1: Tables S3 and S4. The MAE range of Model A was 0.121178–0.125245 and RMSE range was 0.162079–0.176927. The range of MAE was 0.120612–0.126302 and RMSE varied from 0.160373 to 0.172766 for Model B. The OLS Model B was the best mapping model with the lowest MAE and the RMSE (MAE = 0.120612, RMSE = 0.160373). Table 3 shows the regression coefficients from OLS Model B with the whole data of training set. Remainder items and physical * emotion were significant variables in OLS Model B.

**Table 2:** Fitting results from tenfold cross-validation

	Model A		Model B	
	MAE	RMSE	MAE	RMSE
OLS	0.121178	0.164604	0.120612	0.160373
Tobit	0.122966	0.162079	0.120697	0.162394
MM	0.123800	0.176927	0.124862	0.172766
Clad	0.125245	0.168051	0.126302	0.167214

**Table 3:** Regression coefficient and standard error of the optimal mapping model

	Coefficient (SE)
Constant	0.9575 (0.0591)^#^
Physical	− 0.0066 (0.0037)
Emotion	− 0.0091 (0.0058)
Remainder items	− 0.0039 (0.0017)^#^
Physical^2^	0.0001 (0.0001)
Emotion^2^	0.0006 ((0.0004))
Physical * emotion	− 0.0009 (0.0003)^#^
Age	− 0.0014 (0.0008)
Sex (women)	− 0.0009 (0.0187)

^#^*P* < 0.05

### Testing results

**Fig. 2:**
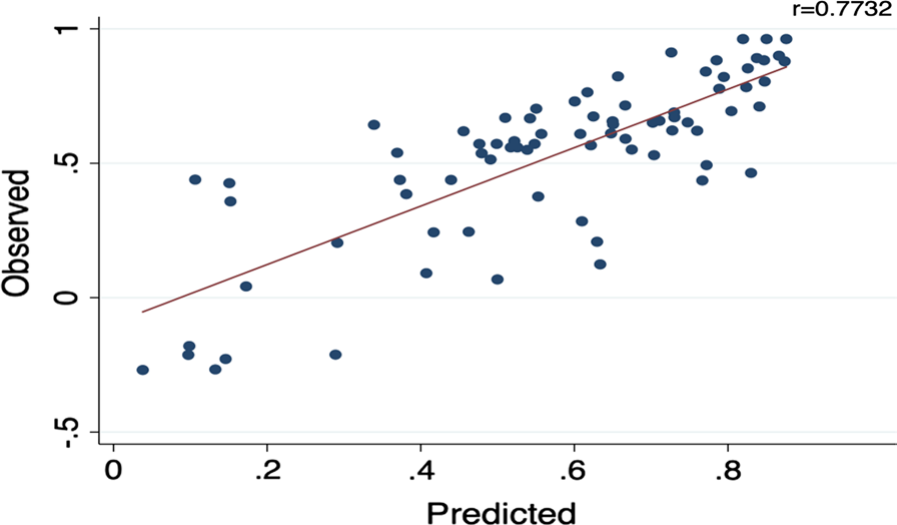
The observed versus predicted SF-6Dv2 mapped from the optimal mapping model

**Fig. 3:**
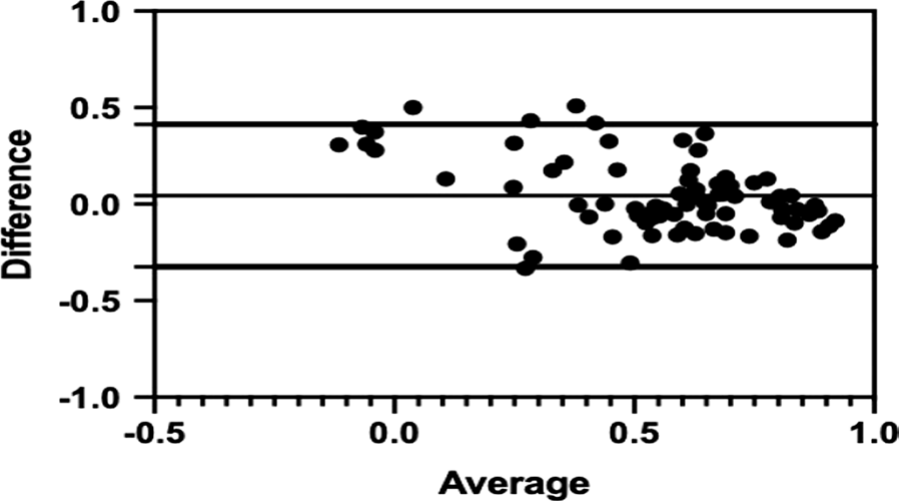
Bland and Altman plot of differences between the observed and the predicted SF-6Dv2 value of OLS Model B

As shown in Fig. 2, the observed and predicted values of SF-6Dv2 are positively correlated with OLS Model B in the testing set. Spearman correlation test result showed that the predicted value was highly correlated with the observed value (r = 0.7732). The X axis of the Bland–Altman plots shows the average of the predicted and observes SF-6Dv2 value, the Y axis is the distribution of differences between the predicted and observed SF-6Dv2 value. The Bland–Altman plots shows that there is the better fitting effective in the better health states, and the model will overvalue the SF-6Dv2 utility in poor health states (Fig. 3). The specific predicted results are shown in Table 4. Observed 25th, 50th and 75th percentile were 0.436, 0.600 and 0.7110 while predicted values were 0.464, 0.619 and 0.7600, the prediction errors fell in a range of 0.01–0.05. But there was a major prediction error for observed minimum value.

**Table 4:** Fitting results of testing set with the optimal mapping model

	Mean	Minimum	Maximum	P.25	Median	P.75	MAE	RMSE	ICC
Observed	0.531	− 0.269	0.962	0.436	0.600	0.7110	0.142828	0.192225	0.743
Predicted	0.575	0.038	0.876	0.464	0.619	0.7600			
Abs diff	0.044	0.307	0.086	0.028	0.019	0.049			

Abs diff: The absolute value of the difference between predicted utility and observed utility

## Discussion

For the measurement of health utility of patients with CHF, EQ-5D is currently the most commonly used measuring instrument [28–30]. SF-6Dv1 was developed later than EQ-5D, and was expanded along with the widely use of SF-36 in the world. Now the application of SF-6Dv1in the world is becoming more widely acceptable [16, 31, 32], and is the one of most widely used instrument in economic evaluation [33]. EQ-5D-5L can describe up to 3125 health states [34] with obvious ceiling effect, while SF-6Dv1can describe more than 18,000 health states with higher sensitivity and lower upper limit effect than EQ-5D-5L [31, 35]. SF-6Dv1 also has a certain floor effect [6] and the sensitivity of SF-6Dv1 will decrease when the health state deteriorates. Hence professors [36] proposed that SF-6Dv1 may play an import role for conditions which are mild, such as relatively stable heart failure patients. SF-6Dv2 was developed in 2020 considering the criticisms of SF-6Dv1, and it improved the validity of psychometric tests and increased the ability to measure change in health status over time [25]. In this study, the mapping algorithms were adopted to transform the information of MLHFQ instrument into the health utility value of SF-6Dv2, which could reduce the burden of patients and obtain the health utility data related to disease, thus providing richer data for health economic evaluation.

In authors’ knowledge, there are two international studies with MLHFQ as the starting instrument [37, 38], EQ-5D-5L and the assessment of quality of life 8D (AQOL-8D) as the mapping instrument respectively, and the research samples were all from Australia. So far, there has been no study on MLHFQ as the starting instrument for Chinese population. In this study, the training set constructed eight alternative models (two models and four statistical algorithms) with tenfold cross-validation technique. According to the MAE and RMSE, OLS Model B was finally selected as the best mapping model, which was consistent with the best mapping algorithm determined by Catchpool et al. [37] (MLHFQ mapping to AQOL-8D). Yousefi et al. [39] mapped QLQ-C30 onto SF-6Dv2 using the data from colorectal and breast cancer patients in a developing country, the best mapping algorithm was also OLS.

In this study, the scoring range of MLHFQ in baseline sample and follow-up sample almost covered the information of the MLHFQ instrument. The cardiac function classification of NYHA in the full sample (baseline and follow-up) had a nice distribution. These indicated that the sample is a good representation for CHF. There was a high negative correlation between the total MLHFQ score and the observed utility of SF-6Dv2 in the full smaple (r = − 0.8187), and the correlation coefficient was slightly lower than that of Catchpool et al. [37] (r = − 0.825), but higher than that of Kularatna et al. [38] (r = − 0.580). The physical domain scores, emotion domain scores and remainder items scores of MLHFQ also highly correlated with SF-6Dv2 in the full sample. These high correlation indicated that the information of MLHFQ could convert to SF-6Dv2. The MAE and RMSE were lower than the mapping study about the MLHFQ mapped to EQ-5D-5L [38], and similar to the study about MLHFQ mapped to AQOL-8D [37]. The predicted utility values of the optimal models established were highly correlated with the observed values, indicating that the model constructed in this study had a good predictive efficiency and could be applied to cost utility analysis of cardiovascular diseases such as CHF in China.

There was a better goodness of fit when the health state was good according to the Bland–Altman plots and scatter plots. When the health state was deteriorated, the predicted health utility values were not stable and were often overestimated. Similar results can be seen in the study mapped QLQ-C30 ontoSF-6Dv2 [39]. So when the value of SF-6Dv2 increased, the fitting effect were better. Therefore, the application of mapping algorithms from MLHFQ to SF-6Dv2 introduced in this study may be more suitable for the CHF patients with better health state. For CHF patients with worse health state, the researchers should be cautious to obtain health utility value by the OLS algorithm.

This study was not without its limitations. Firstly, the sample size of the study is not big. In order to reduce the impact of this limitation, tenfold cross-validation technique was employed. Although similar sample size can be conducted in the other mapping studies [36, 37]. Bigger sample size is still recommended in future mapping study, and further verify the mapping algorithm which were used in this study. Secondly, although our sample included various stage CHF patients, there were less NYHA I and IV patients. Therefore, the results should be used cautiously for NYHA I and IV patients.

## Conclusion

To the authors’ knowledge, this is the first mapping algorithm which convert MLHFQ to SF-6Dv2. The results of this study suggested that MLHFQ can be mapped to SF-6Dv2, and the information of MLHFQ can be used to obtain appropriate health utility values for the economic evaluation of health management strategies and therapeutic interventions for CHF or other related cardiovascular diseases.

## Supplementary Information

**Additional file 1. Supplemental data.** Mapping the Minnesota Living with Heart Failure Questionnaire (MLHFQ) to SF-6Dv2 in Chinese patients with heart failure.

## Data Availability

The datasets used during the study are available from the corresponding author on reasonable request.

## Abbreviations

MLHFQ: Minnesota Living with Heart Failure Questionnaire
OLS: Ordinary least square
MM: Robust MM estimator
CLAD: Censored least absolute deviations
MAE: Mean absolute error
RMSE: Root mean square error
ICC: Intraclass Correlation Coefficients
HF: Heart failure
QALYs: Quality adjusted life years
NICE: National Institute for Health and Clinical Excellence
CHF: Chronic heart failure
NYHA: New York Heart Association
SF-36: Short Form-36
Abs diff: The absolute value of the difference between predicted utility and observed utility
AQOL-8D: Assessment of quality of life 8D
