# Supplementary material for: Mapping the Minnesota Living with Heart Failure Questionnaire (MLHFQ) to SF-6Dv2 in Chinese patients with heart failure
Source: Health Qual Life Outcomes. 2022 Jun 20;20:98. doi: 10.1186/s12955-022-02004-x (PMC9208129; doi:10.1186/s12955-022-02004-x)
Supplement: Supplementary file 1 — Additional file 1. Supplemental data. Mapping the Minnesota Living with Heart Failure Questionnaire (MLHFQ) to SF-6Dv2 in Chinese patients with heart failure. [file 12955_2022_2004_MOESM1_ESM.docx]

**Mapping the Minnesota Living with Heart Failure Questionnaire (MLHFQ) to SF-6Dv2 in Chinese patients with heart failure**

Supplemental data

Table S1 The differences between baseline and follow-up samples

|  | MLHFQ | Physical | Emotion | SF-6Dv2 |
| --- | --- | --- | --- | --- |
| *Z* | -6.267 | -7.165 | -4.823 | -6.143 |
| *P* | 0.000 | 0.000 | 0.000 | 0.000 |

Table S2 The correlation between dimensions of SF-6Dv2 and MLHFQ

|  | Total | Physical | Emotion | Remainder items |
| --- | --- | --- | --- | --- |
| Physical function | -0.7022 | -0.6929 | -0.5679 | -0.6390 |
| Role function | -0.7652 | -0.6929 | -0.6377 | -0.7112 |
| Social function | -0.6161 | -0.6065 | -0.5736 | -0.5226 |
| Pain | -0.4535 | -0.4196 | -0.4964 | -0.3841 |
| Mental health | -0.5583 | -0.4726 | -0.6528 | -0.4771 |
| Vitality | -0.6814 | -0.6598 | -0.6068 | -0.6009 |
| SF-6Dv2 utility | -0.8187 | -0.7836 | -0.7637 | -0.7192 |

Table S3 Goodness-of-Fit results from10-fold cross-validation on Model A

|  |  | MAE |  |  |  |  | RMSE |  |  |
| --- | --- | --- | --- | --- | --- | --- | --- | --- | --- |
|  | OLS | Tobit | MM | CLAD |  | OLS | Tobit | MM | CLAD |
| Fold-1 | 0.104565 | 0.121599 | 0.107555 | 0.147550 |  | 0.176710 | 0.166743 | 0.171448 | 0.172340 |
| Fold-2 | 0.108970 | 0.116475 | 0.130358 | 0.105012 |  | 0.181473 | 0.228456 | 0.155420 | 0.115647 |
| Fold-3 | 0.126875 | 0.158808 | 0.110958 | 0.131082 |  | 0.189310 | 0.149626 | 0.174631 | 0.129294 |
| Fold-4 | 0.117846 | 0.129981 | 0.082865 | 0.078510 |  | 0.135976 | 0.169736 | 0.202199 | 0.165961 |
| Fold-5 | 0.114696 | 0.126265 | 0.119621 | 0.106685 |  | 0.129947 | 0.191328 | 0.207746 | 0.201450 |
| Fold-6 | 0.119360 | 0.124797 | 0.128867 | 0.184296 |  | 0.157910 | 0.168905 | 0.161524 | 0.141713 |
| Fold-7 | 0.132169 | 0.116303 | 0.104358 | 0.121974 |  | 0.124725 | 0.119511 | 0.143037 | 0.174091 |
| Fold-8 | 0.125303 | 0.105961 | 0.143583 | 0.122628 |  | 0.174343 | 0.179440 | 0.190021 | 0.187785 |
| Fold-9 | 0.157373 | 0.119576 | 0.176520 | 0.134628 |  | 0.162501 | 0.140391 | 0.149763 | 0.194212 |
| Fold-10 | 0.104621 | 0.121599 | 0.133313 | 0.120083 |  | 0.213149 | 0.106651 | 0.213477 | 0.198014 |
| average | 0.121178 | 0.122966 | 0.123800 | 0.125245 |  | 0.164604 | 0.162079 | 0.176927 | 0.168051 |

Table S4 Goodness-of-Fit results from10-fold cross-validation on Model B

|  |  | MAE |  |  |  |  | RMSE |  |  |
| --- | --- | --- | --- | --- | --- | --- | --- | --- | --- |
|  | OLS | Tobit | MM | CLAD |  | OLS | Tobit | MM | CLAD |
| Fold-1 | 0.119338 | 0.124203 | 0.111661 | 0.099645 |  | 0.157175 | 0.176135 | 0.131364 | 0.154416 |
| Fold-2 | 0.117195 | 0.094491 | 0.168464 | 0.136270 |  | 0.176709 | 0.153187 | 0.207156 | 0.166573 |
| Fold-3 | 0.132258 | 0.144343 | 0.087459 | 0.146115 |  | 0.201657 | 0.143602 | 0.180880 | 0.180459 |
| Fold-4 | 0.099600 | 0.123864 | 0.169633 | 0.117605 |  | 0.147457 | 0.160811 | 0.112016 | 0.145421 |
| Fold-5 | 0.120952 | 0.133081 | 0.146595 | 0.133111 |  | 0.111728 | 0.151070 | 0.172741 | 0.171675 |
| Fold-6 | 0.134124 | 0.116373 | 0.109060 | 0.132088 |  | 0.127466 | 0.167582 | 0.192330 | 0.144104 |
| Fold-7 | 0.110648 | 0.123561 | 0.098880 | 0.119757 |  | 0.187977 | 0.156641 | 0.147028 | 0.172638 |
| Fold-8 | 0.119821 | 0.123307 | 0.168944 | 0.124256 |  | 0.174465 | 0.185209 | 0.221190 | 0.174924 |
| Fold-9 | 0.135580 | 0.120027 | 0.107577 | 0.152364 |  | 0.160542 | 0.186712 | 0.186568 | 0.176264 |
| Fold-10 | 0.116609 | 0.103722 | 0.080350 | 0.101805 |  | 0.158554 | 0.142994 | 0.176387 | 0.185664 |
| average | 0.120612 | 0.120697 | 0.124862 | 0.126302 |  | 0.160373 | 0.162394 | 0.172766 | 0.167214 |
